# Antimicrobial Effects of Plant-Based Supplements on Gut Microbial Diversity in Small Ruminants

**DOI:** 10.3390/pathogens13010031

**Published:** 2023-12-29

**Authors:** Ian K. Daniel, Obadiah M. Njue, Yasser M. Sanad

**Affiliations:** 1 Department of Agriculture, University of Arkansas at Pine Bluff, Pine Bluff, AR 71601, USA; 2 Department of Veterinary Pathobiology, School of Veterinary Medicine & Biomedical Sciences, Texas A&M University, College Station, TX 77843, USA; 3 Department of Epidemiology, University of Arkansas for Medical Sciences, Little Rock, AR 72205, USA

**Keywords:** pre-slaughter intervention, microbial diversity, antimicrobial resistance, microbiome, dietary supplements

## Abstract

Every year in the United States, approximately 48 million people are affected by bacterial illnesses that are transmitted through food, leading to 3000 fatalities. These illnesses typically stem from food animals and their by-products, which may harbor dangerous pathogens like *Salmonella enterica*, *Listeria monocytogenes*, enterohemorrhagic *Escherichia coli* O157:H7, and *Campylobacter jejuni*. Factors that contribute to contamination include manure used as a soil amendment, exposure to polluted irrigation water, and contact with animals. To improve food safety, researchers are studying pre-slaughter intervention methods to eliminate bacterial contamination in live animals. While small ruminants are vital to global agriculture and income generation for small farms, traditional feeding practices involve supplements and antibiotics to boost performance, which contributes to antibiotic resistance. Hence, researchers are looking for friendly bacterial strains that enhance both animal and human health without impacting livestock productivity. The global trend is to minimize the use of antibiotics as feed supplements, with many countries prohibiting or limiting their use. The aim of this review is to provide a comprehensive insight on the antioxidant capabilities, therapeutic attributes, and applications of bioactive compounds derived from sweet potato tops (SPTs), rice bran (RB) and radish tops (RTs). This overview provides an insight on plant parts that are abundant in antioxidant and prebiotic effects and could be used as value-added products in animal feed and pharmaceutical applications. This review was based on previous findings that supplementation of basal diets with natural supplements represents a multifaceted intervention that will become highly important over time. By remarkably reducing the burden of foodborne pathogens, they apply to multiple species, are cheap, do not require withdrawal periods, and can be applied at any time in food animal production.

## 1. Introduction

**Figure 1:**
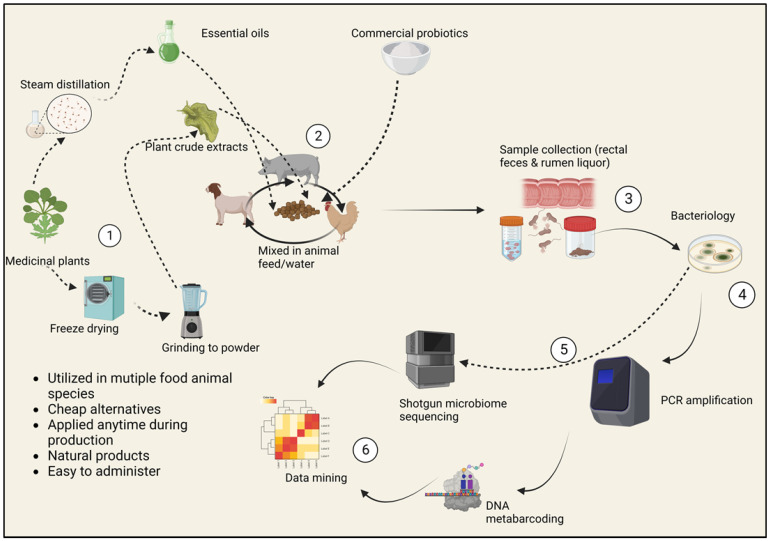
The selection of “traditional” food safety interventions before slaughter incorporates emerging food safety issues, including antibiotic replacement, genome sequencing of microbiomes and cost–benefit analysis. (1) Preparation of plant-derived supplements. Extraction involves a steam distillation process in the case of essential oils or grinding into powder for crude extracts. (2) Supplements may be mixed in feed or drinking water for easy administration. (3) Fecal and ruminal sample collection. (4) Morphological identification of bacteria, (5) Molecular identification of bacteria and genome sequencing of marker genes. (6) Microbiome data analysis.

Every year, approximately 48 million people in the United States fall sick due to illnesses caused by foodborne bacteria, 128,000 are hospitalized, and 3000 others die. Common important foodborne disease agents in the United States food sector include *Campylobacter jejuni*, enterohemorrhagic *Escherichia coli O157:H7*, *Salmonella enterica* (non-typhoidal), *Clostridium perfringens*, norovirus, *Staphylococcus aureus* and *Listeria monocytogenes* [1,2,3,4]. Food animals and their products are the primary sources of these illnesses [5]. The gastrointestinal tract of food animals is a potent natural habitat for these opportunistic microbes [6]. A group of pathogenic microbial species travel the intestinal path asymptomatically; however, their expulsion in vast amounts can occur through defecation and then transmission by other vectors to agricultural produce and humans [7]. Most of the contamination is accelerated by using manure as a soil amendment, the use of polluted irrigation water and the contact of produce and animals [8]. The closeness of the human population to poultry and other food animals as well as pet animals facilitates the host–host transfer of enteric microbiota, which means that a “healthy microbiome” is necessary to block these transmissions, which lead to foodborne illnesses [9]. In highly populated areas, the complexity and diversity of food animals’ microbiome is a remarkable tool that can be exploited to mitigate the carriage of the bad bacteria that impact the safety of animal products [7]. Therefore, the current review aims to discuss how various pre-slaughter intervention approaches can be applied in the live animal to help eradicate bacterial contamination. In small ruminants, pre-harvest interventions are multifactorial practices involving the improvement of animal health and productivity, convenience, and the cost-effective analysis of their applications. The selection of “traditional” food safety interventions before slaughter incorporates emerging food safety issues, including antibiotic replacement, genome sequencing of microbiomes and cost–benefit analysis (Figure 1). These insights are supported by the work of Eli Metchnikoff, the Russian Nobel Prize winner who proposed that the gut microbiota is subject to manipulation owing to its dependence on diet. Hence, harmful pathogens can be replaced with beneficial microbes to improve the health of humans and animals and ultimately food safety [10].

On the other hand, small ruminants play a vital role in the income generation and food supply for small farm systems and global agriculture. Feed supplements and veterinary antibiotics have continually been used to enhance livestock farming and improve small ruminant performance. Among the feed supplements are feed antibiotics, which have taken a huge role for their utilization as growth promoters in animal diets [11]. The use of antibiotics in any setting contributes to the growing global threat of antimicrobial resistance, calling for strategic plans to preserve antibiotic efficacy while concurrently upholding food safety and security [12,13]. This means control of unnecessary use of antibiotics while discovering alternate ways to prevent infections. Livestock microbiota is assuming a focal point of concern for microbiologists, veterinarians, and animal nutrition laboratories in efforts to identify friendly bacterial strains with probiotic properties [10] There have been escalating discussions regarding microbial resistance to antimicrobials, the control and termination of antibiotic use as feed supplements, and consumer decline in demand for antibiotic-based foods. In 2010 alone, the total utilization of antibiotic drugs in food animals was estimated at 63,151 tons and escalating [12]. Significant knowledge has been gained through culture- and PCR-based techniques to obtain a more in-depth insight into thinner groups of bacteria or genomes, for example, erythromycin opposition in swine fecal isolates [14]. In fact, in the European Union, the consumption of antimicrobial growth promoters (AGPs) in the production of food animals was phased out in 2006, while in the US, the Food and Drug Administration (FDA) strongly restricted AGPs in the animal sector in December 2016. Health Canada addressed the same measures in December 2017, and this trend is anticipated in many countries across the world [15].

## 2. Burden of Pathogens in Small Ruminant Source Foods

Foodborne illnesses reported from meat and meat product sources account to a great economic and public health safety concern [16]. Like many other animal source foods, such as eggs, poultry meat, pork, beef, and dairy products, small ruminant source foods in the United States continue to present potential food safety risks [17]. Foodborne pathogenic bacteria can be acquired from sheep and goat meat, although small ruminants account for relatively fewer cases compared to other meat sources [17]. Unlike a few decades ago, with the increasing production of small ruminants for meat and milk production, the association of goat and sheep meat with foodborne diseases is likely to grow more frequently. The health of meat goats and sheep is highly dependent on hygienic housing conditions, proper nutrition, and fencing; however, even with these factors addressed, perfectly healthy animals are not guaranteed [18]. Transmission and spread of disease agents occur mainly via two routes: ingestion of contaminated food, after which the disease pathogen establishes by proliferation and colonization of the gut, or the ingestion of bacterial toxins from toxigenic agents by the human host [16]. The ingestion of contaminated food with pathogens causes foodborne infections associated with a longer incubation period, while foodborne intoxications arise from the ingestion of food products with bacterial toxins, which are characterized by a short incubation period [16,19]. The second route of spread involves the direct contact of human skin with animals and their biological substances such as saliva, urine, fecal matter, milk, semen, and blood. This pathway affects meat handlers at slaughterhouses and processing plants as well as individuals in close contact with companion animals [20,21]. Poor hygiene at abattoirs has been reported as an avenue for contamination by pathogenic bacteria, including *Campylobacter*, *Salmonella enterica,* and Shiga toxin producing *E. coli* (STECs). These pathogens may ride in fecal material, inside the GIT or the hides and skin of animals presented for harvest and can be transferred to the carcass during skinning and evisceration. Furthermore, carcass contamination specifically occurs when contaminated materials such as knives, workers hands, skin and fecal material has direct contact with meat during slaughter operations [22,23]. For instance, within the US, Mexico and the Bahamas, a study found a *Salmonella enterica* frequency of 17.1% (*n* = 339) and *E. coli* O157 was detected at 1.5% (*n* = 266) in small ruminant hide surfaces [23]. Another study found the presence of *Salmonella enterica* on the hide and feces of show goats and lambs. While stock show animals are not a primary concern for the meat supply, they ultimately end up being slaughtered [24]. Hygiene and sanitation at abattoirs, livestock shows and exhibitions where small ruminants are in direct contact with people should be emphasized to reduce the risk of pathogen contamination and ultimately on the carcasses during harvesting.

This study focuses on foodborne pathogens from small ruminant (goats and sheep) gastrointestinal tract (GIT) that compromise the health of the public by finding their way into food sources of both animal and plant origin.

## 3. Global Threat of Antimicrobial Resistance

Antimicrobial resistance (AMR) is a phenomenon of antimicrobial drugs becoming ineffective in stopping and killing the bacteria they are designed to kill or stop from multiplying. Multidrug resistance in both human health and veterinary medicine has elevated to disturbing levels in many parts of the globe and is now perceived as a significant rising risk to worldwide public health and food security [25]. Antimicrobial resistance is a global threat, with new mechanisms of antimicrobial resistance spreading across continents at a speedy rate. The World Health Organization (WHO) has declared the AMR scourge a global emergency due to recent evolution of pan-resistant organisms, and it is postulated that if nothing is done by the year 2050, AMR will kill 10 million people annually worldwide outnumbering cancer because the antimicrobials available now would not be effective anymore [26,27,28,29]. One of the significant concerns is that AMR may compromise certain contemporary medical procedures [30] and essential treatments, like surgery and transplants, which heavily rely on antibiotics [31]. Earlier research indicates that the majority of deaths and surgical failures happen because of early posttransplant infections [32]. In addition, treatments like cancer chemotherapy weaken the patient’s immune cells, making them vulnerable to infections. In a global situation where antimicrobial drugs become ineffective, such therapies would become extremely risky and ineffective [33].

## 4. Growing Global Interest in Small Ruminants as a Food Source

Small ruminants, like sheep and goats, are becoming an increasingly popular food source worldwide for various reasons. Their ability to graze on vegetation that is inappropriate for other kinds of livestock and their adaptability to harsh environments means that they are excellent for small-scale farmers in low- and middle-income countries (LMICs). The fact that small ruminants are easier to handle and process than other livestock, like cattle, contributes to the growing interest in them as a food source. This makes them a good choice for small farmers who do not have access to large processing plants. Additionally, organic and sustainable farming methods, which are gaining in popularity all over the world, are ideal for small ruminants.

In general, the adaptability, nutritional value, and sustainability of small ruminants are the driving forces behind global interest in them as a food source. Small ruminants are likely to play an increasingly significant role in meeting these requirements as the world’s population continues to rise and the demand for protein continues to rise. The global small ruminant numbers continue to proliferate, and goat production alone is beyond 1 billion [34]. Arid regions are home to more than 50% of the global small ruminating animals. Among these animals, goats and sheep play a central economic role, especially in the LMICs of Africa, Asia and Oceania [35]. Apart from being sources of domestic and commercial meat, milk and fiber production, small ruminants are regarded as sources of emergency income in these areas [36,37]. Small ruminants are an appealing source of animal protein for human consumption due to their ability to convert plant matter into high-quality protein. The production of dairy goats was estimated to be 218 million globally in 2017, with Asia leading with over 50% production. In the United States, dairy goat production was roughly estimated to be 4% in the same year. In a span of 10 years, the production of dairy goats increased by roughly 22%, yet the major income from global dairy goat farming is generated from the sale of meat and meat products [34]. The International Goat Association (IGA) has been offering supporting goat-related research since 1982 to maximize economic growth, alleviation of world poverty and promote prosperity while enhancing the quality of life. For the past 20 years, the IGA has been expanding its impact and service worldwide by connecting small ruminant research to producers, processors, and markets, mainly by running global conferences on goats [38]. Sheep and goat farming play a significant socioeconomic role, especially in low- and medium-income countries (LMICs); however, it has lately been facing huge threats, such as zoonotic pathogen sources and climate change challenges [39,40].

## 5. Small Ruminant Production in the United States

The demand for goat and sheep meat in the US is currently beyond production, mainly due to the rising settlement of immigrant families. Immigrant ethnicities such as Hispanic, Muslim, Africans, Caribbean, and Asian populations use goat meat for cultural functions and other festivals [41,42]. Prospects for the improved purchasing power of these ethnic families in the future are wonderful and call for expanded goat meat industry [41]. Goats are mainly reared for meat production, but can also be kept for dairy purposes, fiber, and livestock shows, as well as companion animals. Experimental models have also been used to promote clinical studies that evaluate the effect of dairy items on human health. Research has been conducted on the influence of various diets on sheep breeding. This involves feeding on probiotics, essential oils, and improved pasture to boost the nutritional qualities of cheese, fat, and flesh [43]. Maintenance requirements for small ruminants are easily affordable for many farms because small ruminants can be raised with little supplemental feed and fewer land resources. Besides being easy to care for animals, their production is profitable from land that has not been maximally utilized [42]. Organic production is labor- and cost-friendly, with emerging markets anticipated every day. According to the USDA (2011), farmers with fewer than 500 goats make a significant contribution to the nation’s overall goat production [44]. The state of Texas has the most goat farms in the United States, accounting for approximately 36.3 percent of all goats produced [45]. This could be because Texas’s climate and environment are suitable for goat farming.

## 6. The Link between Antimicrobial Use and the Emergence of Antimicrobial Resistant Bacteria in Food Animals

For over six decades, veterinary antimicrobials like aminoglycosides, tetracyclines, fluoroquinolones, glycopeptides, and sulfonamides have been employed in veterinary practice. However, their usage in livestock and humans has led to the development and spread of antimicrobial-resistant bacterial reservoirs [46]. Regardless of being asymptomatic carriers of pathogenic microbiota, food animals, companion animals, and other farm animals are susceptible to infections. Food and companion animals can transfer resistant microbes to their owners and to the public while also spreading pathogens to produce, agricultural environment and facilities increasing the incidence of resistant pathogenic bacteria to humans and act as a reservoir for human disease [47]. The emergence of antimicrobial resistance from commonly used antimicrobials that play a vital role in treating human illnesses, such as fluoroquinolones and third-generation cephalosporins for *S. enterica* and *Campylobacter* infections, has significant implications for public health [48]. In recent times, two factors of prescription antimicrobials have been notable in increasing the risk of resistance selection: the quantity of antimicrobials used and the utilization of doses that are too low or treatments that are excessively prolonged [49].

Recently, there has been escalating concern over the presence of drug residues in foods of animal origin as well as the potential effects of microbiologically active residues of veterinary antimicrobials on the human gut microbiome [50]. The presence of drug residues in muscle foods has been recognized globally, and their detection in live animals, feeds and animal carcasses at slaughter and retail products becomes a critical surveillance stage in residue control [51]. The primary factors related to residual effects of antimicrobials in edible tissues and animal products such as milk are animal age and use, and ignorance regarding withdrawal periods for regular and extra-label uses [52]. Further, antimicrobials in animal production have also been reported to cause drug toxicity and allergic reactions in humans [53,54].

The animal microbiota incorporates numerous nonpathogenic bacteria, but also opportunistic pathogens that may obtain and spread resistance genes inside the microbial network, primarily by horizontal gene transfer (HGT) [55,56]. Due to continued intensive prescription of antimicrobials to food animals, contaminated carcasses may harbor resistant bacteria such as *E. coli.* These antimicrobial resistant bacteria can donate the resistance genes to other commensal and pathogenic strains, putting human medicine into a post-antibiotic world where simple infections become untreatable [57,58]. Diet plays a key role in the ruminal resistome, its pathogenicity and abundance of AMR genes with potential consequences for human and animal well-being [59]. The exposure of foregut (rumen) and hindgut (rectum) microbiota to antimicrobials reveal perturbations within 3 to 14 days, with a long-term imbalance that persists for over 18 days after antimicrobial withdrawal [60]. Another study using 16s *rRNA* gene sequencing investigated the aftereffects of streptomycin injection on ruminal fluid and ruminal mucosa (RM) microbiota in goats in vivo. The results suggested alteration of both microenvironments by inducing increases in the diversity and richness of RM microbiota with significant expansion of *Prevotella*, *Pseudomonas*, *Pedobacter*, and *Flavobacterium*, which are regular members of biofilm communities attached to injured tissue [61]. Antibiotically distracted gastrointestinal microbiota needs an extensive amount of time to be reestablished to the original state, which may create major complications for the host [60]. Additionally, overuse and misuse of veterinary antimicrobials is a focal point in AMR due to selective pressures exerted on pathogenic and commensal bacteria [46,62].

## 7. Examples of Resistant Animal-Borne Bacterial Species

The antimicrobial effects of plant-based supplements on foodborne pathogens have garnered significant attention within the realm of small ruminant gut microbial diversity. Small ruminants, such as sheep and goats, are integral to many agricultural communities worldwide. Understanding and enhancing their gut health is crucial not only for the well-being of the animals but also for ensuring the safety of food products derived from them. Plant-based supplements enriched with bioactive compounds have been explored as potential tools to modulate the gut microbiota of these animals. These supplements exhibit antimicrobial properties against foodborne pathogens, contributing to improved gut health and ultimately enhancing the safety and quality of small ruminant-derived food products. This research underscores the intricate relationship between plant-based supplementation, gut microbial diversity, and the mitigation of foodborne pathogens, offering valuable insights into sustainable and health-conscious small ruminant farming practices.

Numerous studies have been published on the antimicrobial and antioxidant properties of various plant components. However, there is relatively little information on the impact of such components on the enhancement of probiotics and production of antimicrobial compounds from these probiotics against foodborne pathogens.

### 7.1. Salmonella enterica

Non-typhoidal *Salmonella* continues to pose a substantial burden on public health worldwide. In the United States alone, 26,500 hospitalizations and about 420 deaths are recorded annually according to CDC national *Salmonella* surveillance [63]. Specifically, *S. enterica,* a principal cause of morbidity and mortality, remains a tenacious zoonotic pathogen whose control in many parts of the world has been elusive. Over 2600 serotypes of *S. enterica* have been described, many of which demonstrated host specificity [64,65,66]. Both humans and animals, including small ruminants such as goats, are known to get sick from *Salmonella* [67]. After exposure and ingestion, *S. enterica* invades the intestinal epithelial lining of the ileum and colon, facilitating bacterial entry, either to cause neutrophilic gastroenteritis or subsequent damage to systemic sites and sepsis. It thrives in the intracellular niche, allowing intrinsic antimicrobial resistance and chronic colonization in rare cases [68]. Most cases are linked to food as the vehicle of exposure. Ground beef products are highly implicated due to the pathogen’s ability to survive within the lymphatic system and colonize the peripheral lymph nodes [69,70,71]. Consequently, efforts to address human exposure to *S. enterica* have heavily relied on production by regulators and food packers [72]. *S. enterica* is an important pathogen of food-producing animals and can be a problem in intensive farming systems. Producers suffer substantial production losses and animal wastage due to salmonellosis.

### 7.2. Listeria monocytogenes

The bacterial genus *Listeria* comprises 21 species of Gram-positive, motile, facultative anaerobic, non-spore-forming rods up to 2 µm in length [73,74]. Of these, *Listeria monocytogenes* (*L. monocytogenes*) has been extensively studied. *L. monocytogenes* is a facultative intracellular bacterium that can cause severe foodborne infection in humans and invasive diseases in animals, including farm ruminants [75]. In addition to asymptomatic carriage of the microbe, just like human listeriosis, small ruminants can also develop the disease and develop clinical symptoms including neurological and fetal infections [76]. *L. monocytogenes* is widely recognized as a possible microbial contaminant of raw small ruminant milk; however, there is a paucity of data on the presence, risk factors and contamination dynamics and public health data in small ruminant farms [77]. This pathogen is the causative agent of human invasive listeriosis, a disease characterized by severe bloodstream infections, meningitis and death in advanced cases [78]. Baher et al., 2021 reported potential for retailed goat and offal for harboring drug-resistant *Listeria*-related genes using PCR [79]. In addition, Silva-Guedes et al. (2022) found that fresh goat legs harbored antibiotic residues that could affect the meat microbiota, since microbial diversity was lower in samples with residues [79].

### 7.3. Escherichia coli

The foodborne pathogen *Escherichia coli* (*E. coli*), a frequent bacterial cause of foodborne and bloodstream infections worldwide, processes virulent traits that allow invasion, colonization and disease occurrence in humans and animals [80]. As a commensal bacterial species, *E. coli* is ubiquitous in the environment, colonizes the gut of apparently healthy mammalian and avian species, and a commonality of symptoms between humans and animals suggests that *E. coli* organisms are zoonotic [81]. Under susceptible conditions, patients develop symptoms ranging from severe diarrhea to hemolytic uremic syndrome (HUS) following ingestion [82,83]. Among extended-spectrum β-lactamases (ESBLs) producing Gram-negative bacteria, *E. coli* is considered the most abundant in host environments representing both an indicator pathogen and a predominant vehicle for resistance transmission in one health context [84,85]. A study published from Jordan in 2022 by Obadiat et al. reported small ruminants as reservoirs of colistin-resistant and *mcr-1*-positive *E. coli* that exhibit coresistance to critically important antimicrobials [86].

### 7.4. Campylobacter spp.

*Campylobacter* spp., primarily *C. jejuni* and *C. coli*, inhabit the gastrointestinal tract of mammals, including goats, sheep, and chickens [86]. Specifically, the foodborne pathogen *Campylobacter jejuni*, a leading cause of foodborne enteritis, is estimated to account for between 400 and 500 cases of gastroenteritis every year. *C. jejuni* commonly colonizes and invades the intestinal epithelial cells. In susceptible individuals, severe symptoms ranging from fever and severe abdominal cramping to bloody diarrhea manifest [87]. *Campylobacter* spp. have not been extensively studied in small ruminants compared to other food animals (majorly in poultry). However, the pathogen has been isolated from ovine carcasses and liver, intestinal contents and feces [88]. Furthermore, resistant *Campylobacter* has also been isolated from milk samples in sheep and goats in previous studies. Resistant strains have also been reported, especially in sheep isolates, suggesting that *Campylobacter* exposure to antimicrobials used in both human and veterinary medicine exerts strong selection pressures known to promote the emergence and rising prevalence of antimicrobial-resistant *Campylobacter* [89].

### 7.5. Clostridium perfringens

The human and animal enteric pathogen *Clostridium perfringens* has been linked to several human and animal-associated intestinal complications, including food poisoning and preterm necrotic enterocolitis, throughout the past decade. Advances in genomic tools make it an ideal time to reexamine this clinically important human and veterinary pathogen [90,91]. *C. perfringens* is a spore-forming gut bacterium that resides naturally in the soil and intestinal tracts of apparently many healthy warm-blooded animals, including small ruminants. *C. perfringens*-mediated infections are a frequent problem in the food industry due to the ubiquitous nature of the pathogen that plays a role in its ability to grow and survive in foods and pathogenesis [92]. *C. perfringens* genotypes A, B, C, D and E produce lethal toxins that cause enteric diseases such as clostridial abomasitis and enteritis in goats and sheep [91]. In small ruminants, moderate-to-severe enterotoxaemia is usually reported and associated with the pathogen’s adaptability to produce a wide arsenal of toxins. Several studies have reported *C. perfringens* from the feces of both healthy and sick animals (sheep and goats) [93]. For example, a 2018 study by Hamza et al. reported the highest prevalence of *C. perfringens* in apparently healthy sheep (65.45%), followed by goats (58%), buffalo (55%) and cattle (47%) [94]. In addition, other studies demonstrated the presence of multidrug resistance (MDR) patterns for commonly used antimicrobials in food animal production, including β-lactams, tetracyclines and aminoglycosides [95,96,97,98].

## 8. The Ruminant Intestinal Microbiota—The Gut Microbiome

The gastrointestinal microbiome has been proposed to function like a genuine organ [99]. The body’s immunity depends on its ability to regulate various processes such as gut homeostasis and inflammatory response. The balance and stability in the intestinal environment are sustained by the symbiotic relationship between the resident microorganisms and the intestinal tract [6]. A majority of microorganisms in the gastrointestinal tract are beneficial bacteria that work closely with the enterocytes in a symbiotic relationship [99]. The ruminal ecosystem is highly varied in composition. The microbiome of the digestive system of ruminants is a complex and dynamic assembly, with principal inhabitants of the rumen including bacteria, methanogenic archaea, fungi, protozoa, and viruses, including lytic bacteriophages, with direct or indirect functions towards organic matter degradation. The microbial composition of the digestive system of ruminants is estimated to be inhabited by over 5000 species of microorganisms [100]. The rumen is the most numerous and most diversely populated part of the digestive system of ruminants. The second-largest number of microorganisms can be found in the large intestine [100]. The composition of the microbiome depends on factors such as breed, age, external environment, and nutrition. The organisms work in synergy or individually to degrade food material that is indigestible to humans to provide metabolic energy to their host and the release of methane in case of archaea [100]. In the categorization of mammals as monogastric and polygastric, the clustering of their microbial communities occurs in a way that corresponds to those classifications. Polygastric animals such as goats and sheep have a four-stomached digestive tract with the rumen as the largest and most important section. The rumen is filled with billions of tiny fiber-degrading microorganisms that belong to *Ruminococcus*, *Lactobacillus*, *Prevotella*, *Streptococcus*, *Butyrivibrio*, *Megasphaera*, *Bacteroides* and *Fibrobacter* [9]. The highly nutritious protein foods that humans drive from animals are constituted by their ability to use their sophisticated rumen microbiota to process plant cell wall materials that are inedible to humans, without competition for food [100]. Many scientists have generally shifted their attention on studying the rumen microbiome to address the many global challenges of the livestock industry. Microbial communities in the lower gut have an equally profound impact on the host, especially during early life stages. The fetal GI environment is considered sterile until initial exposure in the birth canal [101]. Microbiota acquired during passage through the birth canal has been highlighted in long-term immunity of newborns. The colonization of the rumen at birth depends primarily on vaginal microbiota and milk colostrum to shape the gastrointestinal microbiomes [102,103]. Beyond the rumen, changes in pH, a varying number of secretions and oxidation-reduction potential dictate the structure of the associated microbiome [104].

Previous studies confirmed that most vaginal microbes were found to express similarities to bacteria and methane-producing archaea found in the gut, indicating that gastrointestinal microbial populations play a vital role in developing premature ruminant gut [105]. A study by Shabana et al. showed no significant variations in the composition of the gut bacterial communities of sheep and goats at the same age; however, drastic changes in the diversity of gut bacterial microbiota increase with the animal’s age [106]. In colonic and rectal samples of bacterial diversity in camels, high numbers of *Ruminococcaceae* and *Akkermansia* were detected, while the forestomach and ileum showed a significant colonization of *clostridium* species and *Bacteriodales* [100,107]. Consequently, rumen and rectal samples may not provide an accurate representation of the gut microbiome structure or composition [108], considering the clear differences in the microbial populations of the rumen, rectal feces, and other segments of the gastrointestinal tract. The abomasum and the beginning of the ileum provide a holistic environment for the microbiota due to the presence of complex enzymatic activities and low pH. However, compared to the rumen, the population and diversity of microbiota in this environment decrease significantly by several orders of magnitude [100,109]. The mucosal surface of the gastrointestinal tract is the largest among all mucosal surfaces in the body, boasting a massive epithelial barrier that spans 400 m^2^. This large surface area enables it to effectively detect the invasion of pathogens and to capture viruses, bacteria, and other pathogen-associated molecular patterns. Additionally, it serves as a connection between stimulators of the immune response and the lymphoid tissue located in the gut [110,111,112]. The rumen microbiota has a profound impact on animal health conditions, food safety and production potential.

## 9. Impact of Dietary Supplements on the Gut Microbiome

**Figure 2:**
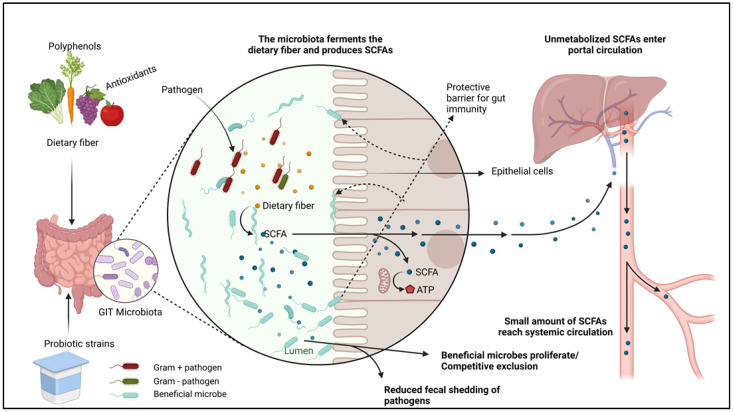
The mechanism of the plant-based supplements as interventions in the gut microbiota when used individually or synergistically with probiotic supplements. Bioactive elements in plant extracts together with probiotics promote the proliferation of beneficial bacteria while suppressing colonization by pathogenic bacteria (e.g., competitive exclusion). Beneficial bacteria enhance gut barrier integrity, promote the degradation of dietary fiber and the overall balance between the intestinal microbiome and produced metabolites, including short-chain fatty acids (SCFAs).

Safe antibacterial alternatives such as herbs have been used since antiquity as natural medicines and food preservatives [113]. These plants play a crucial role in animal agriculture by allowing producers and veterinarians to reduce extensive synthetic antibiotic consumption in food-producing animals [114]. Studies have shown that aromatic plants possess bioactive compounds that stimulate animal health and performance. Some of the noticeable benefits of plant extracts and essential oils include the increased degradation of proteins and fibers, immunity improvement and methanogen reduction. These interventions have been reported to be effective when used individually or synergistically with probiotic supplements (Figure 2) [115]. Essential oils have been shown to be promising feed additives in mitigating methane and ammonia emissions. Positive effects such as growth response and inhibition of pathogenic bacterial growth have been broadly witnessed in poultry, pigs, ruminant food animals and rabbits [116]. New, alternative ways of developing the gut microbiome using prebiotic oligosaccharides and probiotic additives have continued to be explored, showing fascinating potential in nurturing the beneficial commensal bacteria in the intestinal ecosystem [117].

A group of studies has shown that a shift from a normal diet can cause transient shifts in the adult gut microbial community within 24 h, after which the normal state immediately returns. While microbiota dysbiosis can lead to disease susceptibility, immune microbiomes play a vital role in maintaining healthy body functions, and diet is the principal factor in manipulating the gastrointestinal microbiome [118]. Additionally, new ways of developing the gut microbiome using prebiotic oligosaccharides and probiotic additives continue to be explored, showing fascinating potential in nurturing the beneficial commensal bacteria in the intestinal ecosystem [9,118].

Several studies have also confirmed substantial antimicrobial activities of various plant essential oil components against enteropathogenic microbes in food animals. For example, five plant extracts—thyme, sage, laurel, myrtle, and orange oils—exhibited antibacterial and bacteriostatic properties against common foodborne bacteria, such as *Listeria monocytogenes*, *E. coli*, *Candida albicans*, and *S. aureus* [119]. The global essential oil market demand was valued at over 21 billion USD in 2022 and is estimated to increase by a compound annual growth rate (CAGR) of 7.9% from 2023 to 2030. Growing aromatherapy applications, coupled with rising consumption from the personal care and cosmetics industry, are likely to fuel product demand over the forecast period [120]. For example, the orange essential oil segment alone is poised to rise at a steady pace owing to its increasing consumption in the personal care and cosmetics industry [121].

## 10. Antimicrobial Traits of Plant-Based Essential Oils and Other Plant Extracts

Plants and their secondary metabolites, such as volatile oils, have been utilized in folk medicine for an extended period, particularly in countries like China [122]. Nevertheless, medicinal properties including antimicrobial, antioxidant, and anti-inflammatory are extensively investigated for purposes of medicine in the modern world [119,123]. The various capabilities of medicinal plants have been attributed to their creation of secondary metabolites such as essential oils (EOs), terpenoids, polyphenols, tannins, alkaloids, coumarins and flavonoids. Studies have revealed that optimum effectiveness is realized from plant compounds that contain phenolic groups [124,125]. Essential oils or volatile oils are concentrated oily aromatic natural liquids derived from plant material (leaves, wood, roots, flowers, buds, twigs, bark, and seeds), which have been proved to possess bioactive substances with broad medicinal capabilities [126,127]. These EOs are naturally used to give protection against pathogenic bacteria and parasitic organisms [128,129,130]. The antimicrobial traits of plant derivatives are usually controlled by the structure, components and functional groups present in the oil. EO antimicrobial capabilities are attributed to their hydrophobic nature, able to disrupt membrane integrity and the mitochondrial lipid layers. In addition, cell osmotic pressures are drastically altered [124,131]. Although dietary EOs have been associated with antioxidative, antimicrobial, and anti-inflammatory capabilities, their difference in composition, together with the variance of used tests, has revealed a great diversity of results [132]. Several studies have also evaluated the activity of various EO substances on foodborne pathogens found in small ruminant gastrointestinal tracts to ascertain their effects on intestinal morphology, gut flora and performance, patterns of fermentation and product quality. Data available demonstrate their ability to inhibit pathogenic foodborne bacteria such as *Salmonella*, *E. coli*, *Listeria monocytogenes*, *Staphylococcus aureus* and *Shigella dysenteria*, with Gram-positive bacteria showing higher susceptibility compared to Gram-negative bacteria [127,133]. For example, Sun et al. reported positive influences on gut morphology and normal flora structure of Sewa sheep supplemented with 3000 mg/kg EO concentrate [134]. In another study, oregano EO containing cineole (terpenoid oxide) revealed anti-inflammatory and analgesic activity [135]. The quality of lamb products has been improved (reduced off-flavor perception) by addition of garlic or juniper EO in lamb diets, while rancid-odor perception has been reduced in displayed meats [136]. A recent study conducted on goat rumen using metagenomic analysis unveiled that EO–cobalt complexes had a considerable impact on both the structure and function of the rumen microbiota. The analysis highlighted a positive relationship between the presence of *Bacteroides* spp. and *Succinivibrio* spp. and increased production of volatile fatty acids (VFAs) in the groups that received supplementation. Additionally, pathway analysis aimed at predicting functionality indicated an increase in activity within lipid and carbohydrate pathways due to EO–cobalt complexes [137]. Other studies using combinations of 50% timothy grass and 50% concentrate showed enhanced microbial populations by increasing combinations of some oils like thymol and carvacrol and showed highly effective synergistic effects when used at equal proportions of 50% each against several enteric foodborne pathogens [138]. It worth noting that there are no approved methods yet to determine MICs for all these plant-based supplements to measure the antimicrobial effects.

## 11. Examples of Plant-Based Dietary Supplements with Antimicrobial Effects

### 11.1. Sweet Potato (Ipomoea batatas) Tops (Leaves and Vines)

Sweet potato (SP) greens or vegetative parts, a broadly popular diet in Africa and Asia, are considered for efficient production of dietary polyphenols, including phenolic acids and anthocyanins. There are at least 15 known biologically active anthocyanins and 6 polyphenolic compounds with medicinal properties incorporated in SPT. Additionally, the SPT can be considered a reliable source of proteins, ranging from 4.0% to 27.0% in leaves and 1.0% to 9.0% in roots [139]. These amounts are interestingly higher in comparison to other vegetable crops like spinach, tea and grape seed [140], and has demonstrated immunomodulatory, antimicrobial, antifungal and anticarcinogenic properties without detectable toxicity to normally replicating cells, such as those in the gastrointestinal wall and the bone marrow [141,142]. Even though studies on antimicrobial functions of SP leaves and tops in small ruminants are lacking, a few specified reports have concluded that the SP plant contains some degree of antimicrobial activity against various Gram-positive and Gram-negative bacterial strains in other species and in food preservation. Sweet potato contains plenty of valuable phytochemicals, some of which are peculiar to specific varieties. Yellow leaf sweet potatoes (YLSPs) showed the highest concentrations of polyphenolic and flavonoid compounds at 11.293 µg/g and 44.963 µg/g, respectively, in a 2017 study [143]. In another study evaluating the phytochemical composition and antimicrobial properties of the SP leaf, water extract showed the best antimicrobial activity against foodborne pathogens *Salmonella enterica* subsp. *enterica* serovar Typhi and *E. coli* in comparison to peptone and ethanol extracts [144]. Water extracts exhibited the best antimicrobial activity against common bacterial pathogens in comparison to peptone and ethanol extracts [144]. SP growers in the US appear to have focused on the size and grade of the product most demanded by their end users: medium size and number one grade [145]. The less requested sizes (Jumbos and Canners) and the uncommonly used parts such as the vines and leaves, therefore, end up being used back as seed for the next growing season or left in the fields for soil fertility enhancement. Alternative uses for these “market rejects” would be profitable for SP farmers.

### 11.2. Daikon Radish (Raphanus raphanistrum subsp. sativus)

While a pool of in vitro studies has been reported on therapeutic properties of radish defensins, in vivo studies, especially in small ruminants, are scarce. Management practices of cover crop radish are cost-effective and could deliver more profitable practices, however, its use as a medicinal agent have not been well investigated, hence its massive underutilization. Studies have revealed that pickled radish activates polyphenol oxidase that avails novel phenols with antibacterial activity in vitro [146]. A recent study by Lim and colleagues reported that ethanol extracts from *R. raphanistrum* subsp. *sativus* (radish) powder demonstrated the potential of *R. raphanistrum* subsp. *Sativus* (radish) in inhibiting the growth of *Salmonella enteritidis* 110, *Cronobacter sakazakii* KCTC 2949, *Bacillus cereus* ATCC 10876, and *Staphylococcus aureus* ATCC 6538 in antimicrobial action [147]. Findings from a Turkish study reported that red-type *R. sativus* methanolic extracts displayed broad-spectrum antifungal and antibacterial potential against 52 bacterial species, including a wide range of foodborne pathogens [148]. Further, antiurolithiatic and diuretic activities have been reported from aqueous extracts of *R. sativus* bark in a dose-dependent pattern using rat models. The study confirmed potential use in folk medicine for control against urolithiasis [149]. *Raphani semen* (RS), the radish seed, plays a huge traditional medicinal role in China, India and South Korea as a laxative, for constipation and diarrhea prevention, coughs and distending pain, with antitumor, carminative, and anti-inflammatory effects and reduction in oral squamous cell carcinoma [150,151,152,153]. In summary, these studies demonstrate that *R. raphanistrum* subsp. *sativus*, a neglected medicinal plant, should be regarded as a globally significant crop in animal health with potential to contribute to small ruminant medicine, food safety and economic value. In the United States, tillage radish has a space as a winter cover crop [154]. Typically, the cover crop is terminated above ground before flowering, chemically burned down or ploughed under the soil just before the planting of the successive crop or otherwise winterkills at freezing temperatures [154]. In a grazing situation, the tillage radish concurrently provides two benefits: alleviating soil compaction and anthocyanin-rich forage for livestock.

### 11.3. Rice Bran (Oryza sativa L)

The bioactive elements such as antioxidants found in rice bran have been found to harbor immune-enhancing properties including the increment of the gut–mucosal barrier, reduced intestinal pathogen colonization, and proliferation of beneficial gut microbiota [155,156,157]. Research on rice bran effects in small ruminants is scarce and we have only a rudimentary understanding of the food safety impacts in other animal species. To date, there are no comprehensive studies showing the effects of rice bran supplementation on the gut microbiota of goats or sheep. However, focusing on other animal species, research has demonstrated that consumption of the cereal bran portion boosts the production of short-chain fatty acids (SCFAs), which are potent modulators of the gastrointestinal environment [158]. Another study by Yang et al., 2015 shows that rice bran exerts positive gut-modulatory abilities by favoring the growth of diarrhea-reducing probiotic strains such as *Lactobacillus rhamnosus* [159], which have worked against human rotavirus (HRV) and other diarrhea-related pathogens. Rice bran treated with heat-resistant amylase, protease, and hemicellulase enzymes exhibited reduction in occurrences of inflammatory bowel disease (IBD) by modulating the colonic ecosystem and regulating immune cell differentiation [157]. In a separate study, Kumar et al. found that mice consuming 10% and 20% rice bran diets revealed lowered proinflammatory cytokines up to nine days post-infection reduction in *Salmonella* fecal shedding compared to the control group [160]. Sheflin et al., 2015 reported that dietary intervention with heat-stabilized rice bran (HSRB) improved gut microbiota metabolism of healthy adults by increasing branched-chain fatty acids, secondary components of bile and showed an elevation of eight operational taxonomic units (OTUs), including those from the genus *Bifidobacterium* [157]. Collectively, the above studies suggest the advantageous use of rice by-products as biological additives in animal production and other industries. Small ruminants may benefit from rice bran supplementation in feeds for enhanced health, safe food, and to increase the rate of investment (ROI). The stabilized rice bran has a cost advantage due to its abundance and low cost. The functional food attribute of stabilized rice bran has not been adequately focused on as a novel strategy in pre-harvest food safety, yet it is a rich source of protein, natural vitamin B and a potential prebiotic [161]. In the United States, rice bran varieties have been under cultivation by local farmers in rice-growing counties for several years, and therefore tremendous quantities of rice bran are available from rice millers. Antimicrobial properties of rice bran are well researched in other animal species such as poultry. About 40% of the rice harvest goes to waste at the mill due to the rejection of many by-products [162]. Previous investigations revealed pathogen-inhibitory effects of Carlose rice bran, a common product of the USDA–ARS Rice Research Unit, Stuttgart, Arkansas against *Salmonella enterica* subsp. *enterica serovar* Typhimurium [163]. Lakast is a mid-season, long-grain variety with outstanding yield potential and exceptional milling properties, released in 2014 in Arkansas [164]. The prebiotic effects of Lakast rice bran, a variety released in 2014, have been recommended for consideration as biological supplements in livestock feed. Lakast cultivars (pureline and hybrid) used at the Department of Food Science, University of Arkansas significantly reduced the cecal loads of *S. enterica* subsp. *enterica* serovar Typhimurium and other pathogens in broiler cecal microbiota. More diverse microbiota was exhibited in the treatment group [165].

## 12. Conclusions

Several foodborne pathogens in humans have been reported from different countries. Small ruminant species have been regarded as reservoirs for these organisms. Small ruminants, often considered reservoirs for foodborne pathogens, can benefit from the incorporation of natural dietary supplements, which not only mitigate pathogen colonization but also maintain the animals’ welfare and physiological well-being. The antimicrobial effects of plant-based supplements on the gut microbial diversity of small ruminants hold significant promise as a sustainable and cost-effective approach to enhance pre-harvest food safety and reduce the risk of transmitting foodborne pathogens to humans. Options to reduce the risk of infection from meat consumption include pre-harvest food safety methods to reduce pathogen colonization in live animals and decontamination of meat post-slaughter. However, cost-effectiveness and practicality are critical factors to be considered before adopting any pre-harvest strategies for pathogen reduction in live animals and carcasses. Since small ruminants quickly adapt to rural settings and are less energy-demanding compared to large animals, pre-harvest food safety measures will make more economic sense. Natural dietary supplements are cheap and easy to administer, with less or no compromise to animal welfare and physiological status. Overall, considering the increasing demand for safer meat products and the need to reduce the risk of foodborne illnesses, further research and adoption of plant-based supplements in small ruminant farming practices can contribute to both food safety and economic sustainability.

## References

[1] Heredia N., García S. (2018). Animals as sources of food-borne pathogens: A review. Anim. Nutr..

[2] Scallan E., Hoekstra R.M., Angulo F.J., Tauxe R.V., Widdowson M.-A., Roy S.L., Jones J.L., Griffin P.M. (2011). Foodborne illness acquired in the United States—Major pathogens. Emerg. Infect. Dis..

[3] White A.E., Tillman A.R., Hedberg C., Bruce B.B., Batz M., Seys S.A., Dewey-Mattia D., Bazaco M.C., Walter E.S. (2022). Foodborne Illness Outbreaks Reported to National Surveillance, United States, 2009–2018. Emerg Infect Dis.

[4] Gourama H. (2020). Foodborne Pathogens.

[5] Lee H., Yoon Y. (2021). Etiological Agents Implicated in Foodborne Illness World Wide. Food Sci. Anim. Resour..

[6] Pickard J.M., Zeng M.Y., Caruso R., Núñez G. (2017). Gut microbiota: Role in pathogen colonization, immune responses, and inflammatory disease. Immunol. Rev..

[7] Callaway T., Edrington T., Anderson R., Harvey R., Genovese K., Kennedy C., Venn D., Nisbet D. (2008). Probiotics, prebiotics and competitive exclusion for prophylaxis against bacterial disease. Anim. Health Res. Rev..

[8] Doyle M.P., Erickson M.C. (2006). Reducing the carriage of foodborne pathogens in livestock and poultry. Poult. Sci..

[9] Gaggìa F., Mattarelli P., Biavati B. (2010). Probiotics and prebiotics in animal feeding for safe food production. Int. J. Food Microbiol..

[10] Jensen A.P., Bjørnvad C.R. (2019). Clinical effect of probiotics in prevention or treatment of gastrointestinal disease in dogs: A systematic review. J. Vet. Intern. Med..

[11] Wegener H.C. (2003). Antibiotics in animal feed and their role in resistance development. Curr. Opin. Microbiol..

[12] Van Boeckel T.P., Brower C., Gilbert M., Grenfell B.T., Levin S.A., Robinson T.P., Teillant A., Laxminarayan R. (2015). Global trends in antimicrobial use in food animals. Proc. Natl. Acad. Sci. USA.

[13] Roca I., Akova M., Baquero F., Carlet J., Cavaleri M., Coenen S., Cohen J., Findlay D., Gyssens I., Heure O. (2015). The global threat of antimicrobial resistance: Science for intervention. New Microbes New Infect..

[14] Looft T., Johnson T.A., Allen H.K., Bayles D.O., Alt D.P., Stedtfeld R.D., Sul W.J., Stedtfeld T.M., Chai B., Cole J.R. (2012). In-feed antibiotic effects on the swine intestinal microbiome. Proc. Natl. Acad. Sci. USA.

[15] Maron D.F., Smith T.J., Nachman K.E. (2013). Restrictions on antimicrobial use in food animal production: An international regulatory and economic survey. Glob. Health.

[16] Bintsis T. (2017). Foodborne pathogens. AIMS Microbiol..

[17] Li M., Havelaar A.H., Hoffmann S., Hald T., Kirk M.D., Torgerson P.R., Devleesschauwer B. (2019). Global disease burden of pathogens in animal source foods, 2010. PLoS ONE.

[18] Dawson L., Allen J., Olcott B. (2007). Meat goat herd health procedures and prevention. Proceedings of the 22nd Annual Goat Field Day.

[19] Redel H. (2013). Foodborne infections and intoxications. Emerg. Infect. Dis..

[20] Founou L.L., Founou R.C., Essack S.Y. (2016). Antibiotic resistance in the food chain: A developing country-perspective. Front. Microbiol..

[21] Cassini A., Högberg L.D., Plachouras D., Quattrocchi A., Hoxha A., Simonsen G.S., Colomb-Cotinat M., Kretzschmar M.E., Devleesschauwer B., Cecchini M. (2019). Attributable deaths and disability-adjusted life-years caused by infections with antibiotic-resistant bacteria in the EU and the European Economic Area in 2015: A population-level modelling analysis. Lancet Infect. Dis..

[22] Hansson I., Hamilton C., Ekman T., Forslund K. (2000). Carcass quality in certified organic production compared with conventional livestock production. J. Vet. Med. Ser. B.

[23] Hanlon K.E., Miller M.F., Guillen L.M., Echeverry A., Dormedy E., Cemo B., Branham L.A., Sanders S., Brashears M.M. (2018). Presence of *Salmonella* and *Escherichia coli* O157 on the hide, and presence of *Salmonella*, *Escherichia coli* O157 and Campylobacter in feces from small-ruminant (goat and lamb) samples collected in the United States, Bahamas and Mexico. Meat Sci..

[24] Hanlon K., Brashears M., Miller M. (2019). Presence of Salmonella on the Carcass, Hide and Feces of Goats and Lambs from Major Livestock Shows in Texas Collected Over 4 Yr. Meat Muscle Biol..

[25] Wall B., Mateus A., Marshall L., Pfeiffer D., Lubroth J., Ormel H., Otto P., Patriarchi A. (2016). Drivers, Dynamics and Epidemiology of Antimicrobial Resistance in Animal Production.

[26] Otokunefor K., Agbagwa O.E., Oshifade E., Ibezim C.N.E., Azi E. (2023). Antimicrobial Resistance: A Collective Responsibility.

[27] World Health Organization (2014). Antimicrobial Resistance: Global Report on Surveillance.

[28] Tagliabue A., Rappuoli R. (2018). Changing priorities in vaccinology: Antibiotic resistance moving to the top. Front. Immunol..

[29] Boucher H.W. (2020). Bad bugs, no drugs 2002–2020: Progress, challenges, and call to action. Trans. Am. Clin. Climatol. Assoc..

[30] Toner E., Adalja A., Gronvall G.K., Cicero A., Inglesby T.V. (2015). Antimicrobial Resistance Is a Global Health Emergency. Health Secur..

[31] Prestinaci F., Pezzotti P., Pantosti A. (2015). Antimicrobial resistance: A global multifaceted phenomenon. Pathog. Glob. Health.

[32] Kawecki D., Pacholczyk M., Lagiewska B., Sawicka-Grzelak A., Durlik M., Mlynarczyk G., Chmura A. (2014). Bacterial and fungal infections in the early post-transplantation period after liver transplantation: Etiologic agents and their susceptibility. Transplantation Proceedings.

[33] Teoh F., Pavelka N. (2016). How chemotherapy increases the risk of systemic candidiasis in cancer patients: Current paradigm and future directions. Pathogens.

[34] Miller B.A., Lu C.D. (2019). Current status of global dairy goat production: An overview. Asian-Australas. J. Anim. Sci..

[35] Monteiro A.L.G., Faro A.M.C.d.F., Peres M.T.P., Batista R., Poli C.H.E.C., Villalba J.J. (2018). The role of small ruminants on global climate change. Acta Scientiarum. Anim. Sci..

[36] Panel M.M. (2020). Meat, Milk and More: Policy Innovations to Shepherd Inclusive and Sustainable Livestock Systems in Africa.

[37] Guerra M.M.M., De Almeida A.M., Willingham A.L. (2016). An overview of food safety and bacterial foodborne zoonoses in food production animals in the Caribbean region. Trop. Anim. Health Prod..

[38] Foote W. (2004). History and accomplishments of the International Goat Association during the last 20 years. Small Rumin. Res..

[39] Marino R., Atzori A.S., D’andrea M., Iovane G., Trabalza-Marinucci M., Rinaldi L. (2016). Climate change: Production performance, health issues, greenhouse gas emissions and mitigation strategies in sheep and goat farming. Small Rumin. Res..

[40] Sherman D. (2011). The spread of pathogens through trade in small ruminants and their products. Rev. Sci. Et. Tech.-OIE.

[41] Okpebholo F., Kahan T. (2007). Opportunities and challenges for developing small ruminant systems. Caprine Chron. Off. Newsl. Fla. Meat Goat Assoc..

[42] Coffey L. (2006). Meat Goats: Sustainable Production.

[43] Albenzio M., Santillo A., Avondo M., Nudda A., Chessa S., Pirisi A., Banni S. (2016). Nutritional properties of small ruminant food products and their role on human health. Small Rumin. Res..

[44] Animal and Plant Health Inspection Service, Veterinary Services, Center for Epidemiology and Animal Health (2011). Small-Scale U.S. Goat Operations.

[45] Milani F., Wendorff W. (2011). Goat and sheep milk products in the United States (USA). Small Rumin. Res..

[46] Economou V., Gousia P. (2015). Agriculture and food animals as a source of antimicrobial-resistant bacteria. Infect. Drug Resist..

[47] Prescott J.F. (2008). Antimicrobial use in food and companion animals. Anim. Health Res. Rev..

[48] Anderson A.D., Nelson J.M., Rossiter S., Angulo F.J. (2003). Public health consequences of use of antimicrobial agents in food animals in the United States. Microb. Drug Resist..

[49] Teale C. (2002). Antimicrobial resistance and the food chain. J. Appl. Microbiol..

[50] Piñeiro S.A., Cerniglia C.E. (2021). Antimicrobial drug residues in animal-derived foods: Potential impact on the human intestinal microbiome. J. Vet. Pharmacol. Ther..

[51] Reig M., Toldrá F. (2008). Veterinary drug residues in meat: Concerns and rapid methods for detection. Meat Sci..

[52] Moreno L., Lanusse C. (2017). Veterinary drug residues in meat-related edible tissues. New Aspects of Meat Quality.

[53] Singh S., Shukla S., Tandia N., Kumar N., Paliwal R. (2014). Antibiotic Residues: A Global Challenge. Pharma Sci. Monit..

[54] Beyene T. (2016). Veterinary drug residues in food-animal products: Its risk factors and potential effects on public health. J. Vet. Sci. Technol..

[55] Cameron A., McAllister T.A. (2016). Antimicrobial usage and resistance in beef production. J. Anim. Sci. Biotechnol..

[56] Shterzer N., Mizrahi I. (2015). The animal gut as a melting pot for horizontal gene transfer. Can. J. Microbiol..

[57] Hammerum A.M., Heuer O.E. (2009). Human health hazards from antimicrobial-resistant Escherichia coli of animal origin. Clin. Infect. Dis..

[58] Meek R.W., Vyas H., Piddock L.J.V. (2015). Nonmedical uses of antibiotics: Time to restrict their use?. PLoS Biol..

[59] Auffret M.D., Dewhurst R.J., Duthie C.-A., Rooke J.A., John Wallace R., Freeman T.C., Stewart R., Watson M., Roehe R. (2017). The rumen microbiome as a reservoir of antimicrobial resistance and pathogenicity genes is directly affected by diet in beef cattle. Microbiome.

[60] Ji S., Jiang T., Yan H., Guo C., Liu J., Su H., Alugongo G.M., Shi H., Wang Y., Cao Z. (2018). Ecological Restoration of Antibiotic-Disturbed Gastrointestinal Microbiota in Foregut and Hindgut of Cows. Front. Cell. Infect. Microbiol..

[61] Shen H., Lu Z., Xu Z., Shen Z. (2018). Antibiotic pretreatment minimizes dietary effects on reconstructure of rumen fluid and mucosal microbiota in goats. MicrobiologyOpen.

[62] Lammie S.L., Hughes J.M. (2016). Antimicrobial resistance, food safety, and one health: The need for convergence. Annu. Rev. Food Sci. Technol..

[63] Aljahdali N.H., Sanad Y.M., Han J., Foley S.L. (2020). Current knowledge and perspectives of potential impacts of Salmonella enterica on the profile of the gut microbiota. BMC Microbiol..

[64] Singh V. (2013). Salmonella serovars and their host specificity. J. Vet. Sci. Anim. Husb..

[65] Jajere S.M. (2019). A review of Salmonella enterica with particular focus on the pathogenicity and virulence factors, host specificity and antimicrobial resistance including multidrug resistance. Vet. World.

[66] Bäumler A., Fang F.C. (2013). Host specificity of bacterial pathogens. Cold Spring Harb. Perspect. Med..

[67] Hempstead S.C., Gensler C.A., Keelara S., Brennan M., Urie N.J., Wiedenheft A.M., Marshall K.L., Morningstar-Shaw B., Lantz K., Fedorka-Cray P.J. (2022). Detection and molecular characterization of Salmonella species on US goat operations. Prev. Vet. Med..

[68] Popa G.L., Papa M.I. (2021). Salmonella spp. Infection—A continuous threat worldwide. Germs.

[69] Webb H.E., Brichta-Harhay D.M., Brashears M.M., Nightingale K.K., Arthur T.M., Bosilevac J.M., Kalchayanand N., Schmidt J.W., Wang R., Granier S.A. (2017). Salmonella in peripheral lymph nodes of healthy cattle at slaughter. Front. Microbiol..

[70] Haneklaus A.N., Harris K.B., Griffin D.B., Edrington T.S., Lucia L.M., Savell J.W. (2012). Salmonella prevalence in bovine lymph nodes differs among feedyards. J. Food Prot..

[71] Gragg S.E., Loneragan G.H., Brashears M.M., Arthur T.M., Bosilevac J.M., Kalchayanand N., Wang R., Schmidt J.W., Brooks J.C., Shackelford S.D. (2013). Cross-sectional study examining Salmonella enterica carriage in subiliac lymph nodes of cull and feedlot cattle at harvest. Foodborne Pathog. Dis..

[72] Besser J.M. (2018). Salmonella epidemiology: A whirlwind of change. Food Microbiol..

[73] Quereda J.J., Leclercq A., Moura A., Vales G., Gómez-Martín Á., García-Muñoz Á., Thouvenot P., Tessaud-Rita N., Bracq-Dieye H., Lecuit M. (2020). Listeria valentina sp. nov., isolated from a water trough and the faeces of healthy sheep. Int. J. Syst. Evol. Microbiol..

[74] Schoder D., Pelz A., Paulsen P. (2023). Transmission Scenarios of Listeria monocytogenes on Small Ruminant On-Farm Dairies. Foods.

[75] de Noordhout C.M., Devleesschauwer B., Angulo F.J., Verbeke G., Haagsma J., Kirk M., Havelaar A., Speybroeck N. (2014). The global burden of listeriosis: A systematic review and meta-analysis. Lancet Infect. Dis..

[76] Cardenas-Alvarez M.X., Zeng H., Webb B.T., Mani R., Muñoz M., Bergholz T.M. (2022). Comparative Genomics of Listeria monocytogenes Isolates from Ruminant Listeriosis Cases in the Midwest United States. Microbiol. Spectr..

[77] Condoleo R., Giangolini G., Chiaverini A., Patriarca D., Scaramozzino P., Mezher Z. (2020). Occurrence of Listeria monocytogenes and Escherichia coli in raw sheep’s milk from farm bulk tanks in Central Italy. J. Food Prot..

[78] Hazards E.P.o.B., Ricci A., Allende A., Bolton D., Chemaly M., Davies R., Fernández Escámez P.S., Girones R., Herman L., Koutsoumanis K. (2018). Listeria monocytogenes contamination of ready-to-eat foods and the risk for human health in the EU. EFSA J..

[79] Baher W., Shalaby M., Abdelghfar S. (2021). Prevalence of multidrug-resistant Listeria monocytogenes in retailed goat meat and offal. Damanhour J. Vet. Sci..

[80] Smith S., Wang J., Fanning S., McMahon B.J. (2014). Antimicrobial resistant bacteria in wild mammals and birds: A coincidence or cause for concern?. Ir. Vet. J..

[81] Bélanger L., Garenaux A., Harel J., Boulianne M., Nadeau E., Dozois C.M. (2011). Escherichia coli from animal reservoirs as a potential source of human extraintestinal pathogenic *E. coli*. FEMS Immunol. Med. Microbiol..

[82] Havelaar A.H., Van Duynhoven Y.T.H.P., Nauta M.J., Bouwknegt M., Heuvelink A.E., De Wit G.A., Nieuwenhuizen M.G.M., Van De Kar N.C.A.J. (2004). Disease burden in The Netherlands due to infections with Shiga toxin-producing *Escherichia coli* O157. Epidemiol. Infect..

[83] Boyer O., Niaudet P. (2011). Hemolytic uremic syndrome: New developments in pathogenesis and treatment. Int. J. Nephrol..

[84] Bajaj P., Singh N.S., Virdi J.S. (2016). Escherichia coli β-Lactamases: What Really Matters. Front. Microbiol..

[85] Awosile B., Fritzler J., Levent G., Rahman M.K., Ajulo S., Daniel I., Tasnim Y., Sarkar S. (2023). Genomic Characterization of Fecal Escherichia coli Isolates with Reduced Susceptibility to Beta-Lactam Antimicrobials from Wild Hogs and Coyotes. Pathogens.

[86] Obaidat M.M., Tarazi Y.H., AlSmadi W.M. (2023). Sheep and goats are reservoirs of colistin resistant Escherichia coli that co-resist critically important antimicrobials: First study from Jordan. J. Food Saf..

[87] Ruiz-Palacios G.M. (2007). The Health Burden of Campylobacter Infection and the Impact of Antimicrobial Resistance: Playing Chicken. Clin. Infect. Dis..

[88] Denis M., Rose V., Nagard B., Thépault A., Lucas P., Meunier M., Benoit F., Wilhem A., Gassilloud B., Cauvin E. (2023). Comparative Analysis of Campylobacter jejuni and C. coli Isolated from Livestock Animals to C. jejuni and C. coli Isolated from Surface Water Using DNA Sequencing and MALDI-TOF. Pathogens.

[89] Zhang Q., Beyi A.F., Yin Y. (2023). Zoonotic and antibiotic-resistant Campylobacter: A view through the One Health lens. One Health Adv..

[90] Kiu R., Hall L.J. (2018). An update on the human and animal enteric pathogen Clostridium perfringens. Emerg. Microbes Infect..

[91] Simpson K.M., Callan R.J., Van Metre D.C. (2018). Clostridial abomasitis and enteritis in ruminants. Vet. Clin. Food Anim. Pract..

[92] García S., Heredia N. (2011). Clostridium perfringens: A Dynamic Foodborne Pathogen. Food Bioprocess. Technol..

[93] Mohiuddin M., Iqbal Z., Siddique A., Liao S., Salamat M.K.F., Qi N., Din A.M., Sun M. (2020). Prevalence, genotypic and phenotypic characterization and antibiotic resistance profile of Clostridium perfringens type A and D isolated from feces of sheep (*Ovis aries*) and goats (*Capra hircus*) in Punjab, Pakistan. Toxins.

[94] Hamza D., Dorgham S.M., Elhariri M., Elhelw R., Ismael E. (2018). New insight of apparently healthy animals as a potential reservoir for Clostridium perfringens: A public health implication. J. Vet. Res..

[95] Mohamed H., Elfeky M.M., Al-Azeem A., Wasel F.A. (2023). Molecular characterization of Clostridium perfringens in small ruminants. SVU-Int. J. Vet. Sci..

[96] Khan M.A., Bahadar S., Ullah N., Ullah S., Shakeeb U., Khan A.Z., Khan I.U., Kalhoro N.H., Shah M.B., Malik M.I.U. (2019). Distribution and antimicrobial resistance patterns of Clostridium Perfringens isolated from vaccinated and unvaccinated goats. Small Rumin. Res..

[97] Yadav J.P., Kaur S., Dhaka P., Vijay D., Bedi J.S. (2022). Prevalence, molecular characterization, and antimicrobial resistance profile of Clostridium perfringens from India: A scoping review. Anaerobe.

[98] Hao H., Cheng G., Iqbal Z., Ai X., Hussain H.I., Huang L., Dai M., Wang Y., Liu Z., Yuan Z. (2014). Benefits and risks of antimicrobial use in food-producing animals. Front. Microbiol..

[99] Dudek-Wicher R.K., Junka A., Bartoszewicz M. (2018). The influence of antibiotics and dietary components on gut microbiota. Gastroenterol. Rev./Przegląd Gastroenterol..

[100] Huws S.A., Creevey C.J., Oyama L.B., Mizrahi I., Denman S.E., Popova M., Muñoz-Tamayo R., Forano E., Waters S.M., Hess M. (2018). Addressing global ruminant agricultural challenges through understanding the rumen microbiome: Past, present, and future. Front. Microbiol..

[101] Willyard C. (2018). Baby’s first bacteria. Nature.

[102] Li B., Zhang K., Li C., Wang X., Chen Y., Yang Y. (2019). Characterization and comparison of microbiota in the gastrointestinal tracts of the goat (*Capra hircus*) during preweaning development. Front. Microbiol..

[103] Zhang K., Li B., Guo M., Liu G., Yang Y., Wang X., Chen Y., Zhang E. (2019). Maturation of the goat rumen microbiota involves three stages of microbial colonization. Animals.

[104] Kim Y.-H., Nagata R., Ohkubo A., Ohtani N., Kushibiki S., Ichijo T., Sato S. (2018). Changes in ruminal and reticular pH and bacterial communities in Holstein cattle fed a high-grain diet. BMC Vet. Res..

[105] Bi Y., Cox M.S., Zhang F., Suen G., Zhang N., Tu Y., Diao Q. (2019). Feeding modes shape the acquisition and structure of the initial gut microbiota in newborn lambs. Environ. Microbiol..

[106] Shabana I.I., Albakri N.N., Bouqellah N.A. (2021). Metagenomic investigation of faecal microbiota in sheep and goats of the same ages. J. Taibah Univ. Sci..

[107] He J., Yi L., Hai L., Ming L., Gao W., Ji R. (2018). Characterizing the bacterial microbiota in different gastrointestinal tract segments of the Bactrian camel. Sci. Rep..

[108] Vickers N.J. (2017). Animal communication: When i’m calling you, will you answer too?. Curr. Biol..

[109] Yeoman C.J., Ishaq S.L., Bichi E., Olivo S.K., Lowe J., Aldridge B.M. (2018). Biogeographical differences in the influence of maternal microbial sources on the early successional development of the bovine neonatal gastrointestinal tract. Sci. Rep..

[110] Hallstrom K., McCormick B.A. (2011). Salmonella interaction with and passage through the intestinal mucosa: Through the lens of the organism. Front. Microbiol..

[111] MacDonald T.T., Monteleone G. (2005). Immunity, inflammation, and allergy in the gut. Science.

[112] Turner J.R. (2009). Intestinal mucosal barrier function in health and disease. Nat. Rev. Immunol..

[113] Christaki E., Bonos E., Giannenas I., Florou-Paneri P. (2012). Aromatic plants as a source of bioactive compounds. Agriculture.

[114] Caroprese M., Ciliberti M.G., Albenzio M. (2020). Application of aromatic plants and their extracts in dairy animals. Feed Additives.

[115] Perumalla A., Hettiarachchy N.S., Ricke S.C. (2011). Current perspectives on probiotics in poultry preharvest food safety. Direct-Fed Microbials and Prebiotics for Animals: Science and Mechanisms of Action.

[116] Cobellis G., Yu Z., Forte C., Acuti G., Trabalza-Marinucci M. (2016). Dietary supplementation of *Rosmarinus officinalis* L. leaves in sheep affects the abundance of rumen methanogens and other microbial populations. J. Anim. Sci. Biotechnol..

[117] Simitzis P.E. (2017). Enrichment of Animal Diets with Essential Oils—A Great Perspective on Improving Animal Performance and Quality Characteristics of the Derived Products. Medicines.

[118] Voreades N., Kozil A., Weir T.L. (2014). Diet and the development of the human intestinal microbiome. Front. Microbiol..

[119] Cowan M.M. (1999). Plant products as antimicrobial agents. Clin. Microbiol. Rev..

[120] Irshad M., Subhani M.A., Ali S., Hussain A. (2020). Biological importance of essential oils. Essent. Oils-Oils Nat..

[121] Wilson R. (2002). Aromatherapy: Essential Oils for Vibrant Health and Beauty.

[122] Van Wyk B.-E., Wink M. (2018). Medicinal Plants of the World.

[123] Khamees A.H. (2017). Phytochemical and pharmacological analysis for seeds of two varieties of Iraqi Raphanus sativus. Int. J. Pharm. Sci. Rev. Res..

[124] Celikel N., Kavas G. (2008). Antimicrobial properties of some essential oils against some pathogenic microorganisms. Czech J. Food Sci..

[125] Holley R.A., Patel D. (2005). Improvement in shelf-life and safety of perishable foods by plant essential oils and smoke antimicrobials. Food Microbiol..

[126] Burt S. (2004). Essential oils: Their antibacterial properties and potential applications in foods—A review. Int. J. Food Microbiol..

[127] Man A., Santacroce L., Iacob R., Mare A., Man L. (2019). Antimicrobial Activity of Six Essential Oils Against a Group of Human Pathogens: A Comparative Study. Pathogens.

[128] Nazzaro F., Fratianni F., De Martino L., Coppola R., De Feo V. (2013). Effect of essential oils on pathogenic bacteria. Pharmaceuticals.

[129] Bakkali F., Averbeck S., Averbeck D., Idaomar M. (2008). Biological effects of essential oils—A review. Food Chem. Toxicol..

[130] Adame-Gallegos J.R., Andrade-Ochoa S., Nevarez-Moorillon G.V. (2016). Potential Use of Mexican Oregano Essential Oil against Parasite, Fungal and Bacterial Pathogens. J. Essent. Oil Bear. Plants.

[131] Dorman H.D., Deans S.G. (2000). Antimicrobial agents from plants: Antibacterial activity of plant volatile oils. J. Appl. Microbiol..

[132] Miguel M.G. (2010). Antioxidant and anti-inflammatory activities of essential oils: A short review. Molecules.

[133] Trombetta D., Castelli F., Sarpietro M.G., Venuti V., Cristani M., Daniele C., Saija A., Mazzanti G., Bisignano G. (2005). Mechanisms of antibacterial action of three monoterpenes. Antimicrob. Agents Chemother..

[134] Sun J., Cheng Z., Zhao Y., Wang Y., Wang H., Ren Z. (2022). Influence of increasing levels of oregano essential oil on intestinal morphology, intestinal flora and performance of Sewa sheep. Ital. J. Anim. Sci..

[135] Santos F., Rao V. (2000). Antiinflammatory and antinociceptive effects of 1, 8-cineole a terpenoid oxide present in many plant essential oils. Phytother. Res..

[136] Vasta V., Luciano G. (2011). The effects of dietary consumption of plants secondary compounds on small ruminants’ products quality. Small Rumin. Res..

[137] Lei Z., Zhang K., Li C., Jiao T., Wu J., Wei Y., Tian K., Li C., Tang D., Davis D.I. (2019). Ruminal metagenomic analyses of goat data reveals potential functional microbiota by supplementation with essential oil-cobalt complexes. BMC Microbiol..

[138] Guarda A., Rubilar J.F., Miltz J., Galotto M.J. (2011). The antimicrobial activity of microencapsulated thymol and carvacrol. Int. J. Food Microbiol..

[139] Bovell-Benjamin A.C. (2007). Sweet potato: A review of its past, present, and future role in human nutrition. Adv. Food Nutr. Res..

[140] Sun H., Mu B., Song Z., Ma Z., Mu T. (2018). The in vitro antioxidant activity and inhibition of intracellular reactive oxygen species of sweet potato leaf polyphenols. Oxidative Med. Cell. Longev..

[141] Karna P., Gundala S.R., Gupta M.V., Shamsi S.A., Pace R.D., Yates C., Narayan S., Aneja R. (2011). Polyphenol-rich sweet potato greens extract inhibits proliferation and induces apoptosis in prostate cancer cells in vitro and in vivo. Carcinogenesis.

[142] Islam S. (2006). Sweetpotato (*Ipomoea batatas* L.) leaf: Its potential effect on human health and nutrition. J. Food Sci..

[143] Dewijanti I.D., Banjarnahor S.D., Triyuliani T., Maryani F., Meilawati L. (2017). Antioxidant activity, phenolic and flavonoids total of ethanolic extract of Ipomoea batata L. leaves (white, yellow, orange, and purple). AIP Conference Proceedings.

[144] Mbaeyi-Nwaoha I.E., Emejulu V.N. (2013). Evaluation of Phytochemical Composition and Antimicrobial Activity of Sweet Potato (*Ipomoea batatas*) Leaf. Pak. J. Nutr..

[145] Horton M., Robbins J. (2007). The Potential of the Arkansas Sweet Potato Industry: A Matter of Volatility. J. Bus. Adm. Online.

[146] Li J., Huang S.-Y., Deng Q., Li G., Su G., Liu J., Wang H.-M.D. (2020). Extraction and characterization of phenolic compounds with antioxidant and antimicrobial activities from pickled radish. Food Chem. Toxicol..

[147] Lim H.-W., Song K.-Y., Chon J.-W., Jeong D., Seo K.-H. (2019). Antimicrobial Action of *Raphanus raphanistrum* subsp. *sativus* (radish) Extracts against Foodborne Bacteria Present in Various Milk Products: A Preliminary Study. J. Milk. Sci. Biotechnol..

[148] Kaymak H.C., Yilmaz S.O., Ercisli S., Guvenc I. (2018). Antibacterial Activities of Red Colored Radish Types (*Raphanus sativus* L.). Rom. Biotechnol. Lett..

[149] Vargas S.R., Perez G.R., Perez G.S., Zavala S.M., Perez G.C. (1999). Antiurolithiatic activity of Raphanus sativus aqueous extract on rats. J. Ethnopharmacol..

[150] Ahn K., Ji H., Kim H.-E., Cho H., Sun Q., Shi S., He Y., Kim B.-G., Kim O. (2020). Raphanus sativus L. Seed Extracts Induce Apoptosis and Reduce Migration of Oral Squamous Cell Carcinoma KB and KBC^D133+^ Cells by Downregulation of β-Catenin.

[151] Kang H., Lee S., Eom H., Yu J., Lee S. (2015). Anti-inflammatory and antitumor phenylpropanoid sucrosides from the seeds of Raphanus sativus. Planta Medica.

[152] Gutiérrez R.M., Perez R.L. (2004). *Raphanus sativus* (Radish): Their Chemistry and Biology. Sci. World J..

[153] Choi K.-C., Cho S.-W., Kook S.-H., Chun S.-R., Bhattarai G., Poudel S.B., Kim M.-K., Lee K.-Y., Lee J.-C. (2016). Intestinal anti-inflammatory activity of the seeds of Raphanus sativus L. in experimental ulcerative colitis models. J. Ethnopharmacol..

[154] Roberts T., Ortel C., Hoegenauer K., Wright H., Durre T. (2018). Understanding Cover Crops.

[155] Henderson A.J., Ollila C.A., Kumar A., Borresen E.C., Raina K., Agarwal R., Ryan E.P. (2012). Chemopreventive properties of dietary rice bran: Current status and future prospects. Adv. Nutr..

[156] Ravichanthiran K., Ma Z.F., Zhang H., Cao Y., Wang C.W., Muhammad S., Aglago E.K., Zhang Y., Jin Y., Pan B. (2018). Phytochemical profile of brown rice and its nutrigenomic implications. Antioxidants.

[157] Komiyama Y., Andoh A., Fujiwara D., Ohmae H., Araki Y., Fujiyama Y., Mitsuyama K., Kanauchi O. (2011). New prebiotics from rice bran ameliorate inflammation in murine colitis models through the modulation of intestinal homeostasis and the mucosal immune system. Scand. J. Gastroenterol..

[158] Pham T., Savary B.J., Teoh K., Chen M.-H., McClung A., Lee S.-O. (2017). In vitro fermentation patterns of rice bran components by human gut microbiota. Nutrients.

[159] Yang F., Chen H., Gao Y., An N., Li X., Pan X., Yang X., Tian L., Sun J., Xiong X. (2020). Gut microbiota-derived short-chain fatty acids and hypertension: Mechanism and treatment. Biomed. Pharmacother..

[160] Kumar A., Henderson A., Forster G.M., Goodyear A.W., Weir T.L., Leach J.E., Dow S.W., Ryan E.P. (2012). Dietary rice bran promotes resistance to Salmonella enterica serovar Typhimurium colonization in mice. BMC Microbiol..

[161] Herfel T., Jacobi S., Lin X., Van Heugten E., Fellner V., Odle J. (2013). Stabilized rice bran improves weaning pig performance via a prebiotic mechanism. J. Anim. Sci..

[162] Bodie A.R., Micciche A.C., Atungulu G.G., Rothrock Jr M.J., Ricke S.C. (2019). Current trends of rice milling byproducts for agricultural applications and alternative food production systems. Front. Sustain. Food Syst..

[163] Rubinelli P.M., Kim S.A., Park S.H., Roto S.M., Nealon N.J., Ryan E.P., Ricke S.C. (2017). Differential effects of rice bran cultivars to limit Salmonella Typhimurium in chicken cecal in vitro incubations and impact on the cecal microbiome and metabolome. PLoS ONE.

[164] Moldenhauer K., Sha X., Berger G., Hardke J., Norman R., Wilson Jr C., Wamishe Y., Cartwright R., Blocker M., McCarty D. (2014). LaKast, a high yielding, very short season, long-grain rice variety. BR Wells Rice Res. Stud.-Ark. Agric. Exp. Stn. Univ. Ark. Syst..

[165] Kim S.A., Rubinelli P.M., Park S.H., Ricke S.C. (2018). Ability of Arkansas LaKast and LaKast Hybrid Rice Bran to Reduce Salmonella Typhimurium in Chicken Cecal Incubations and Effects on Cecal Microbiota. Front. Microbiol..

